# Narrow-Band-Imaging-Derived Mean Optical Intensity: A Potential Biomarker for Monitoring the Progression of Oral Squamous Cell Carcinoma

**DOI:** 10.3390/biomedicines14061234

**Published:** 2026-05-29

**Authors:** Zhuwei Huang, Yuan Wang, Yixian Luo, Zixu Zhang, Jiaxuan Huang, Shixian Zang, Pei Ye, Qiao Peng, Ting Liu, Wenmei Wang, Xiang Wang, Ning Duan

**Affiliations:** Nanjing Stomatological Hospital, Affiliated Hospital of Medical School, Institute of Stomatology, Nanjing University, Nanjing 210008, Chinazangshixian715@163.com (S.Z.);

**Keywords:** narrow-band imaging, oral squamous cell carcinoma, mean optical intensity, early diagnosis, dynamic monitoring, quantitative biomarker

## Abstract

**Background/Objectives**: This study aimed to explore the potential value of narrow-band-imaging (NBI)-derived mean optical intensity (MOI) in monitoring the progression of oral squamous cell carcinoma (OSCC), from the normal oral mucosa through epithelial dysplasia to invasive carcinoma. We compared differences in the NBI MOI among distinct pathological stages, so as to provide preliminary evidence for its clinical application in auxiliary diagnosis and progression assessment for OSCC. **Methods**: A total of 40 human oral mucosal specimens (15 normal, 15 oral leukoplakia, 10 OSCC) were enrolled for NBI image acquisition and MOI measurements. A 4-nitroquinoline-1-oxide (4NQO)-induced mouse OSCC model (*n* = 34) was used to dynamically record MOI changes across different pathological stages. A syngeneic tongue tumor mouse model (*n* = 16) was further established to evaluate whether MOI could reflect tumor formation and growth. All MOI values were quantified using ImageJ software with standardized region-of-interest (ROI) selection and background correction. **Results**: In clinical samples, MOI values decreased progressively from the normal mucosa (129.6 ± 5.991 arbitrary units (a.u.)) to oral leukoplakia (OLK) subgroups, including mild dysplasia (104.6 ± 3.757 a.u.) and moderate-to-severe dysplasia (91.77 ± 4.345 a.u.), and further to OSCC (54.41 ± 14.40 a.u.). In the 4NQO model, the MOI of the lingual mucosa was highest in the healthy control group (167.3 ± 10.05 a.u.) and gradually declined with increasing dysplasia severity, reaching the lowest level at the OSCC stage (48.67 ± 10.07 a.u.). In the syngeneic tumor model, the MOI was significantly lower in tumor-bearing mice than in healthy controls (47.85 ± 10.44 a.u. vs. 119.7 ± 14.20 a.u., *p* < 0.001). Receiver operating characteristic (ROC) analysis demonstrated good diagnostic performance of the MOI in distinguishing healthy tissue from cancerous lesions. **Conclusions**: NBI-derived MOI may quantitatively reflect the dynamic alterations of the oral mucosa during oral carcinogenesis and could represent a potential biomarker enabling the non-invasive, repeatable early evaluation and dynamic monitoring of OSCC.

## 1. Introduction

Oral squamous cell carcinoma (OSCC) is the most invasive malignant tumor of the oral and maxillofacial region, with an increasing incidence year by year and a trend towards a younger age of onset [1]. The occurrence and development of OSCC follow a continuous pathological process of normal oral mucosa—epithelial dysplasia—invasive carcinoma [2]. The therapeutic efficacy is closely correlated with the clinical stage of the disease. Early-stage patients undergoing surgical resection have a 5-year survival rate of 70–90%, whereas advanced-stage patients with tumor invasion of adjacent tissues or lymph node metastasis only have a 5-year survival rate of 20–40% after treatment [3]. This significant difference in prognosis highlights the crucial importance of early diagnosis during OSCC progression.

However, the clinical diagnosis of oral potentially malignant disorders and OSCC still faces numerous challenges. Currently, the conventional diagnostic method for OSCC mainly relies on pathological biopsy [4]. Although pathological biopsy is regarded as the gold standard for OSCC diagnosis, it is an invasive procedure that may cause trauma to the oral mucosa. Moreover, it has limitations such as poor repeatability, inability to dynamically monitor lesion progression, and difficulty in sampling small or occult lesions [5]. Other diagnostic methods, including toluidine blue staining, autofluorescence imaging, and chemiluminescence, exhibit deficiencies in consistency and accuracy for early detection [6]. Therefore, exploring non-invasive, efficient, and repeatable diagnostic technologies capable of identifying early lesions throughout the entire course of OSCC has become an urgent clinical need and research hotspot in the field of diagnosis and treatment.

As an emerging optical diagnostic technology, narrow-band imaging (NBI) has shown broad application prospects in the early diagnosis of various mucosa-related tumors, including those of the esophagus, colon, stomach, and oral cavity [7,8,9,10]. Unlike traditional white-light imaging, NBI does not require special contrast agents. Instead, it filters out broadband light to retain two specific narrow-band wavelengths: 415 nm blue light, which corresponds to the main absorption peak of hemoglobin, and 540 nm green light, which facilitates the visualization of blood vessels in the deeper mucosa and submucosa [11]. By capturing tissue surface reflectance signals, NBI enhances endoscopic images and significantly improves the visualization of the mucosal surface architecture and microvascular patterns [12]. This unique optical principle allows NBI to enhance the contrast between mucosal lesions and surrounding normal tissues, clearly displaying subtle changes such as the abnormal microvasculature in early lesions, which are difficult to identify via white-light imaging, thereby providing intuitive visualization evidence for the early identification of tumor lesions [13]. In recent years, relevant studies have confirmed that NBI can significantly improve the early diagnostic rate of head and neck tumors, such as laryngeal cancer and oropharyngeal cancer [14,15]. Nevertheless, in the field of OSCC, existing research on NBI has mostly focused on the qualitative observation of lesion morphology, lacking an in-depth exploration of quantitative indicators that can reflect the biological characteristics of lesions [16,17].

To address the aforementioned research gaps, this study innovatively adopted the NBI-derived mean optical intensity (MOI) as the core quantitative indicator and conducted research from both clinical and animal model perspectives. At the clinical level, NBI was used to image the oral mucosa of patients at different pathological stages, verifying the consistency between this indicator and the pathological diagnosis to clarify its value for early diagnosis. At the animal model level, a 4-nitroquinoline-1-oxide (4NQO)-induced OSCC mouse model was employed to dynamically monitor changes in the indicator throughout the entire process of carcinogenesis to determine the time window for early identification. Additionally, an OSCC syngeneic graft model was combined to evaluate the feasibility of this indicator for tumor growth monitoring and therapeutic response assessments. Ultimately, this study clarifies the diagnostic and monitoring value of NBI MOI during the carcinogenesis process of OSCC.

## 2. Materials and Methods

### 2.1. Subjects and Sample Collection

#### 2.1.1. Clinical Samples

A total of 40 subjects who visited the Department of Oral Mucosal Diseases, Nanjing Stomatological Hospital, from December 2024 to December 2025 were enrolled in this study. NBI was performed on the oral mucosa of all subjects. Based on the clinical diagnosis and histological characteristics, the subjects were divided into three groups: (1) the healthy control (HC) group (*n* = 15), comprising systemically healthy volunteers with a clinically normal oral mucosa and no history of oral mucosal lesions; (2) the oral leukoplakia (OLK) group (*n* = 15), consisting of subjects clinically and pathologically diagnosed with OLK; and (3) the OSCC group (*n* = 10), comprising subjects pathologically confirmed to have OSCC.

The inclusion criteria were as follows: (1) all samples were independently diagnosed by two senior pathologists with consistent diagnostic results; and (2) patients had not received radiotherapy, chemotherapy, or other anti-tumor therapies prior to the examination. Subjects were excluded if they had autoimmune diseases, severe hepatic or renal insufficiency, malignant tumors at other sites, or an inability to cooperate with NBI photography.

The study was approved by the Ethics Committee of Nanjing Stomatological Hospital, Affiliated Hospital of Medical School, Institute of Stomatology, Nanjing University [IRB approval number: 2014NL-002 (KS), 2018NL-008 (KS) and NJSH-2025NL-018]. Prior to enrollment, all subjects were fully informed of the study details and provided written informed consent. Additionally, all image data used for analysis were anonymized.

#### 2.1.2. Animal Samples

Specific-pathogen-free (SPF) C57BL/6 mice (6–8 weeks old, weighing 20–22 g) were purchased from the Experimental Animal Center of Yangzhou University. They were housed in an SPF animal facility under controlled conditions with a temperature of 23 ± 2 °C, relative humidity of 50 ± 5%, and a 12-hour light/12 h dark cycle, with free access to food and water. All mouse experiments were performed in accordance with the Guidelines for Animal Experimentation Ethics. The study was approved by the Animal Ethics and Welfare Committee of Nanjing University (IACUC-D2202108 and IACUC-D2303084).

### 2.2. Main Reagents and Instruments

#### 2.2.1. Reagents

Reagents included 4NQO (purity ≥ 98%, Sigma Aldrich, St. Louis, MO, USA), Dulbecco’s modified eagle medium (DMEM, Gibco, Grand Island, NY, USA), fetal bovine serum (FBS, Gibco); trypsin (Sigma Aldrich), and phosphate-buffered saline (PBS, Solarbio Science & Technology Co., Ltd., Beijing, China).

#### 2.2.2. Instruments

An NBI system (Olympus Corporation, Tokyo, Japan; video processor: OTV-S190, light source: CLV-S190), equipped with 415 nm blue light and 540 nm green light sources, with an image resolution of 1920 × 1080 pixels; a rotary microtome (model RM2235, Leica Microsystems, Wetzlar, Germany); an optical microscope (model CX23, Olympus Corporation); and image analysis software (ImageJ version 1.8.0, National Institutes of Health [NIH], Bethesda, MD, USA) were used.

### 2.3. Experimental Methods

#### 2.3.1. NBI Detection of Clinical Samples

Experienced oral clinicians performed all NBI imaging procedures. Following routine white-light endoscopic examination, images of target lesions and the normal oral mucosa were acquired under low-ambient-light conditions, with a consistent illumination intensity, exposure gain, and white-balance calibration maintained throughout acquisition. The endoscope tip was kept at a fixed working distance from the mucosal surface to ensure reproducible light incidence and uniform magnification. For each sample, images from three distinct visual fields were captured, and at least one high-quality NBI image covering the complete lesion and matched normal mucosa was obtained per case. All images were saved in the original format using identical camera parameters without post-hoc brightness adjustment.

#### 2.3.2. Establishment of 4NQO-Induced OSCC Mouse Model and NBI Monitoring, and Hematoxylin and Eosin Staining

(1) Model establishment: 4NQO was dissolved in sterile drinking water to prepare a 50 μg/mL 4NQO solution. A total of 34 C57BL/6 mice were randomly divided into a model group (*n* = 21) and a control group (*n* = 13). Mice in the model group were given 4NQO-containing drinking water, while those in the control group received regular sterile drinking water for 16 consecutive weeks. After 16 weeks, mice in both groups were switched to normal drinking water and continuously reared until week 24, during which the occurrence of oral mucosal lesions was observed.
(2) Dynamic NBI monitoring: NBI detection of the oral mucosa was performed on mice from both groups at weeks 4, 8, 12, 16, 20, and 24 post-modeling, respectively. Mice were anesthetized via the intraperitoneal injection of 1% sodium pentobarbital (50 mg/kg). The oral cavity was opened with a mouth gag to fully expose the oral mucosa, and the entire dorsal tongue was imaged under white light using the NBI system. After the completion of white-light imaging, the NBI system equipped with dual narrow-band light sources was applied to image the entire dorsal tongue and key target areas (corresponding to the visual fields under white light). The light intensity, exposure parameters, and focal length were kept consistent throughout the imaging procedure for all subjects. Three images of different visual fields were taken for each sample, and all images were preserved in the original format. Subsequently, mice corresponding to five distinct pathological stages were humanely euthanized in compliance with laboratory animal care guidelines. Specifically, an overdose of 1% sodium pentobarbital (150 mg/kg) was intraperitoneally injected to induce rapid, deep anesthesia and the loss of consciousness. A surgical level of anesthesia was confirmed based on the absence of a withdrawal reflex to a noxious stimulus (toe pinch). Upon verification of complete unconsciousness, cervical dislocation was performed as a secondary euthanasia measure to ensure the animal’s death, thereby minimizing unnecessary suffering. Immediately after euthanasia, the entire tongue was dissected using sterile instruments, subjected to ex vivo NBI imaging, and subsequently processed via routine protocols: the tongue specimens were fixed in formalin, dehydrated, embedded in paraffin, and sectioned into 5 μm-thick slices for hematoxylin and eosin (H&E) staining. Histological images were captured using a light microscope (Olympus CX23; Olympus Corporation, Tokyo, Japan), and the resultant slides were scanned with the Panoramic 250 Flash system (3DHISTECH).

#### 2.3.3. Establishment of Mouse OSCC Syngeneic Graft Model and NBI Evaluation

(1) SCC7 cell culture: The mouse SCC7 cell line was purchased from Xiamen ImmunoCell Biotechnology Co., Ltd. (Xiamen, China). Cells were cultured in DMEM supplemented with 10% FBS, 100 U/mL penicillin, and 100 μg/mL streptomycin and maintained in a humidified incubator at 37 °C with 5% CO_2_. Cells in the logarithmic growth phase were harvested for transplantation.
(2) Model establishment: Sixteen C57BL/6 mice were randomly divided into a syngeneic tumor group (*n* = 8) and an HC group (*n* = 8). Mice in the syngeneic tumor group were anesthetized via the intraperitoneal injection of 1% sodium pentobarbital, followed by the subcutaneous injection of a cell suspension (1 × 10^7^ cells/mL, 0.2 mL per mouse) into the right lateral tongue margin. Mice in the control group were injected with an equal volume of sterile DMEM.
(3) NBI monitoring and indicator detection: The volume of syngeneic grafts was measured weekly after modeling, calculated according to the following formula: volume = length × width^2^/2. Meanwhile, NBI detection was performed using the same imaging protocol as described in the 4NQO-induced model section.

#### 2.3.4. Histopathological Evaluation

All histological sections from human clinical specimens and mouse tissues were subjected to a blinded pathological assessment. Briefly, all slides were anonymized with random numerical codes. Two experienced pathologists independently evaluated pathological features without knowledge of sample grouping, clinical information, or the corresponding NBI imaging results. Any diagnostic discrepancies were resolved through consensus discussion.

### 2.4. Image Post-Processing and MOI Measurement

All acquired NBI images were subjected to post-processing and quantitative analysis of the MOI using ImageJ 1.8.0 software. For standardized and reproducible data acquisition, the key procedures were as follows:

(1) Image import and calibration: Raw NBI images in RGB format were imported into ImageJ via File > Open and split into red, green, and blue channels using Image > Color > Split Channels. Green-channel images were selected for subsequent analysis. Optical density calibration was performed by clicking Analyze > Calibrate under the “Uncalibrated OD” preset to unify measurement criteria for images captured in different batches and at different time points. To ensure standardization and reproducibility, consistent background normalization was applied across all samples, with identical calibration parameters used for both clinical and animal datasets to minimize inter-batch variation.
(2) Image preprocessing: For green-channel images, the color balance was adjusted via Image > Adjust > Color Balance to eliminate ambient light interference.
(3) Region of interest (ROI) selection:

Clinical samples: For the patient group, lesion ROIs (ROI_L) were outlined along purple-black/brown optical intensity-reduced areas. For the healthy control group, normal ROIs (ROI_N) of similar sizes were delineated on the corresponding anatomical sites of the oral mucosa in healthy volunteers.

Animal samples: ROI_Ls were accurately outlined to fully cover black-purple lesions in the experimental groups. In the healthy control group, ROI_Ns of similar sizes were selected from corresponding areas of the dorsal tongue, with three non-overlapping regions per sample analyzed to calculate the average MOI.

(4) Background optical intensity correction: Tissue-free areas of similar sizes to ROIs were selected as background references; background values were subtracted from the ROI optical intensities to obtain the corrected MOI, eliminating system background interference.
(5) Optical intensity measurement and data recording: Corrected ROIs were analyzed via Analyze > Measure” to extract the MOI (the core evaluation index). Data were recorded in Excel and expressed as the mean ± standard (SD) deviation for subsequent statistical analysis.
(6) Repeatability verification: The same batch of images was independently measured by two experimenters; the intraclass correlation coefficient (ICC) was calculated, with ICC ≥ 0.85 indicating stable and reliable measurement results.

### 2.5. Heatmap Visualization

(1) Image preprocessing: The preprocessing procedure was consistent with that described in Section 2.4.
(2) Grayscale conversion: Green-channel images were converted to 8-bit grayscale images using Image > Type > 8-bit. The brightness and contrast of all images were uniformly adjusted via Image > Adjust > Brightness/Contrast to ensure comparability of optical density signals across groups.
(3) Standardized pseudo-color lookup table (LUT) application: A unified pseudo-color lookup table (Image > Lookup Tables > Red Hot) was applied to the grayscale images for color mapping. Under this mapping scheme, regions with low optical density (lesional tissues) mainly appeared black, blue, or purple, whereas regions with high optical density (normal oral mucosa) were predominantly orange or yellow, directly reflecting spatial variations in the mucosal optical intensity.
(4) Quantitative color bar generation: To define the quantitative correspondence between pseudo-color and optical density values, a standardized calibration bar was generated on the pseudo-color images using Analyze > Tools > Calibration Bar after parameter adjustment. The color bar was permanently embedded into images and saved via Image > Overlay > Flatten.
(5) Quality control: Identical LUT and image-processing parameters were used for heatmap generation across all images. All operations were independently performed by two researchers to guarantee the reproducibility and the reliability of visualized results.

### 2.6. Statistical Analysis

All experimental data were statistically analyzed using GraphPad Prism 10 (GraphPad Software, Boston, MA, USA) and R 4.3.1 (R Foundation for Statistical Computing, Vienna, Austria). The Shapiro–Wilk test was performed to assess data normality, and normally distributed data were presented as the mean ± SD. The Brown–Forsythe test was used to evaluate homogeneity of variance across groups. For two-group comparisons, an unpaired two-sample Student’s *t*-test was applied. For multi-group comparisons, a one-way analysis of variance (ANOVA) was conducted, followed by Dunnett’s multiple comparison test, using the healthy control group as the reference. Receiver operating characteristic (ROC) analysis was performed with 5-fold cross-validation to mitigate overfitting and optimism bias, reporting the cross-validated area under the curve (cv-AUC), sensitivity, specificity, and corresponding 95% confidence intervals (CIs). The coefficient of determination (R^2^) was calculated to assess how well variations in optical intensity explained changes in the pathological stage. All statistical tests were two-tailed. A *p*-value < 0.05 was considered statistically significant, and *p*-value < 0.0001 was denoted as ****. Violin plots were used for data visualization, incorporating the median, interquartile range, and mean ± SD error bars to intuitively illustrate the distribution of MOIs across different groups.

## 3. Results

### 3.1. Gradual Optical Intensity Loss of Oral Mucosa Under NBI from Normal Mucosa to OLK and OSCC

**Figure 1:**
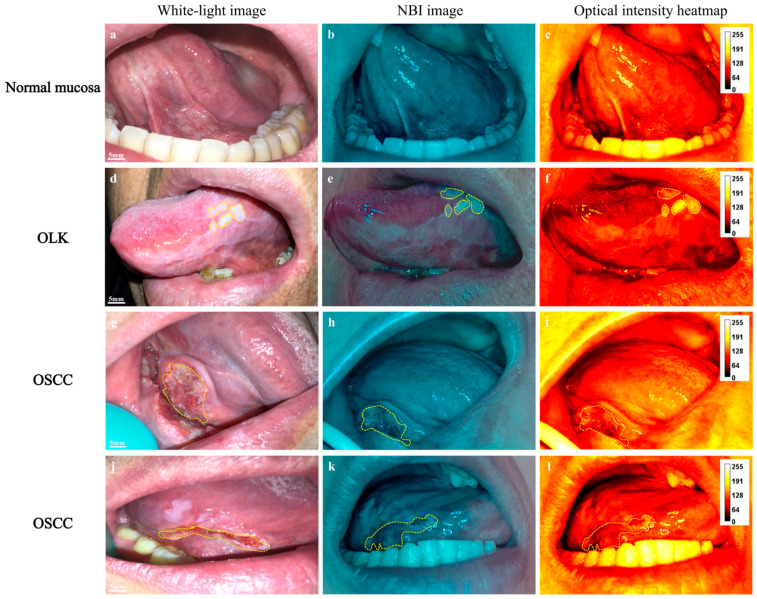
Representative images of normal oral mucosa, OLK, and OSCC under white light, NBI, and optical density heatmap. (**a**,**b**) White-light and NBI images of normal oral mucosa, showing a smooth mucosal surface with a homogeneous optical density distribution; (**c**) corresponding optical density heatmap. (**d**,**e**) White-light and NBI images of OLK lesions, showing thickened, whitish mucosal regions with patchy attenuation of optical density; (**f**) corresponding optical density heatmap with annotated lesion areas. (**g**,**h**,**j**,**k**) White-light and NBI images of OSCC lesions, showing ulcerative/irregular masses with extensive optical density reduction, clearly demarcated from adjacent normal mucosa; (**i**,**l**) corresponding optical density heatmaps with annotated lesion boundaries. Yellow outlines indicate the boundaries of pathological lesions. Scale bar = 5 mm. NBI, narrow-band imaging; OLK, oral leukoplakia; OSCC, oral squamous cell carcinoma.

The NBI system significantly enhances the contrast of superficial and submucosal blood vessels in the oral mucosa by utilizing narrow-band spectra at 415 nm and 540 nm. Combined morphological observation and quantitative analysis in this study demonstrated that the optical characteristics of the oral mucosa under NBI are closely correlated with the pathological nature of lesions. Representative images of each group intuitively revealed that, compared with the normal oral mucosa with a homogeneous optical signal intensity, a localized reduction in optical density was observed in OLK lesions, whereas extensive and marked optical density loss was detected in OSCC lesions (Figure 1). The baseline clinicopathological characteristics of the enrolled subjects are summarized in Table S1.

**Figure 2:**
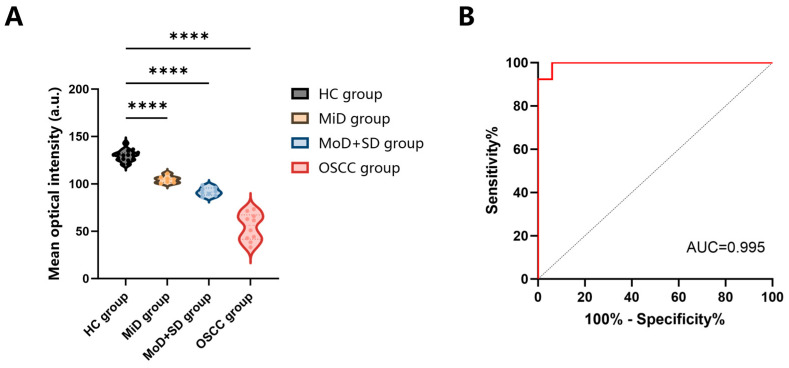
Quantitative analysis and diagnostic performance of MOI across oral mucosal lesions with different pathological grades. (**A**) Violin-box plots showing the stepwise decreasing trend of MOI values in the HC group, MiD group, MoD + SD group, and OSCC group. Data are presented as the mean  ±  SD and were analyzed using one-way ANOVA (**** *p* < 0.0001). (**B**) ROC curve analysis of MOI for distinguishing OSCC from normal oral mucosa and OLK. The red line represents the ROC curve of MOI. AUC = 0.995 (95% CI: 0.983–1.000); stratified 5-fold cv-AUC = 0.962 (95% CI: 0.904–0.993). HC, healthy control; MiD, mild dysplasia; MoD + SD, moderate-to-severe dysplasia; OSCC: oral squamous cell carcinoma; AUC: area under the ROC curve.

Quantitative measurements further confirmed a statistically significant stepwise decreasing trend in MOI from the normal oral mucosa, through mild dysplasia (MiD), moderate-to-severe dysplasia (MoD + SD), to OSCC (HC group: 129.6 ± 5.991 arbitrary units (a.u.); MiD group: 104.6 ± 3.757 a.u.; MoD + SD group: 91.77 ± 4.345 a.u.; OSCC group: 54.41 ± 14.40 a.u., *p* < 0.001) (Figure 2A). This regular variation in optical intensity provides an objective quantitative basis for the non-invasive differentiation of oral mucosal lesions with distinct pathological grades.

To evaluate the clinical value of MOI in distinguishing OSCC, we performed ROC curve analysis (Figure 2B). Using a histopathological diagnosis as the gold standard, we defined OSCC cases as positive and normal mucosa or OLK cases as negative. The results showed that the area under the ROC curve (AUC) of MOI for diagnosing OSCC was 0.9953 (95% CI: 0.9830—1.000), indicating excellent diagnostic accuracy. The optimal cutoff value, determined based on the maximum Youden index (0.97), was 85.73 a.u., yielding a sensitivity of 100% and a specificity of 97.0%. Stratified 5-fold cross-validation was further applied to avoid overfitting and validate the stability of diagnostic performance, yielding a cv-AUC of 0.962 with a 95% CI of 0.904–0.993.

### 3.2. NBI-Derived MOI Is Negatively Correlated with Lesion Severity in 4NQO-Induced Carcinogenesis and Consistent with Pathological Findings

**Figure 3:**
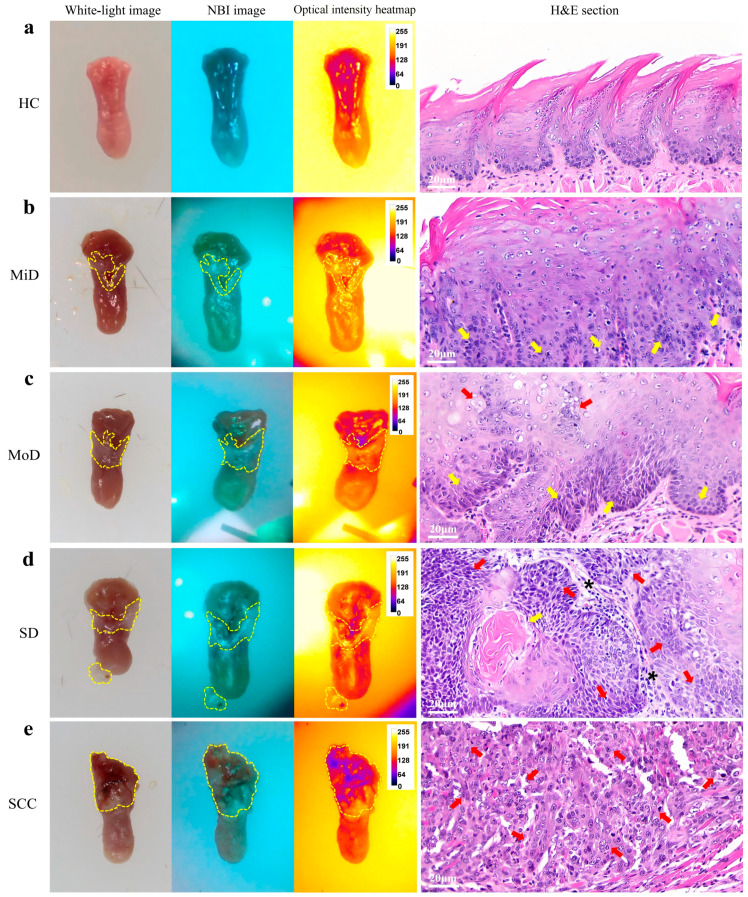
Representative macroscopic, NBI, optical density heatmap, and corresponding H&E-stained histological images of HC, MiD, MoD, SD, and SCC in the 4NQO-induced mouse model. Yellow dashed outlines denote the pathological lesion boundaries in macroscopic and NBI images. (**a**) HC: showing a regular epithelial structure without cellular atypia. (**b**) MiD: architectural disorganization confined to the lower one-third of the epithelium with cellular atypia; yellow arrows indicate proliferative basal-layer cells with abnormal nuclear morphology. (**c**) MoD: epithelial disorganization extending to the middle one-third of the epithelium; yellow arrows indicate proliferative basal-layer cells with nuclear atypia, and red arrows indicate atypical proliferation and abnormal mitoses in spinous-layer cells of the middle epithelial layer. (**d**) SD: epithelial disorganization involving more than two-thirds of the epithelial layer, nearly extending throughout the full epithelial thickness without basement membrane invasion; yellow arrows denote keratin pearls, red arrows indicate loss of basal-cell polarity and markedly increased mitoses, and asterisks indicate an intact basement membrane. (**e**) SCC: red arrows indicate the loss of epithelial stratification, tumor cell invasion into the underlying connective tissue across the basement membrane, pronounced cellular atypia, and increased abnormal mitoses. Scale bar = 20 μm. NBI: narrow-band imaging; HC: healthy control; MiD: mild dysplasia; MoD: moderate dysplasia; SD: severe dysplasia; SCC: squamous cell carcinoma; H&E section: hematoxylin and eosin-stained section.

**Figure 4:**
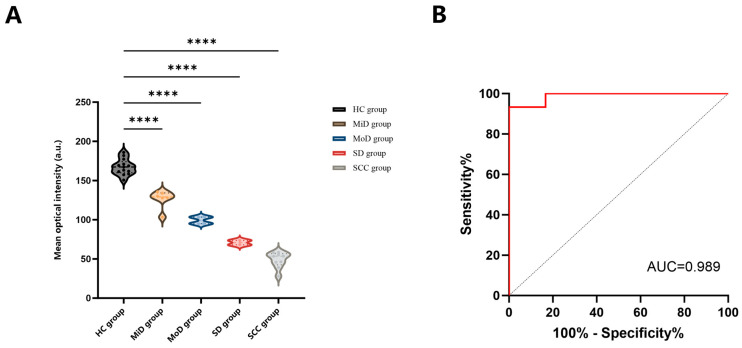
Quantitative comparison and diagnostic performance of MOI across sequential pathological stages of oral lesions in the 4NQO-induced mouse model. (**A**) Violin-box plots showing the stepwise decreasing trend of MOI values in the HC, MiD, MoD, SD, and SCC groups. Significant intergroup differences were assessed via one-way ANOVA (**** *p* < 0.0001). Data are presented as the mean ± SD. (**B**) ROC curve analysis of MOI for discriminating early-stage (MiD) from advanced-stage (MoD, SD, SCC) lesions. The red line represents the ROC curve of MOI. AUC = 0.989 (95% CI: 0.954–1.000). Stratified 5-fold cross-validation further validated diagnostic stability, with a cv-AUC= 0.951 (95% CI: 0.892–0.998). HC, healthy control; MiD, mild dysplasia; MoD, moderate dysplasia; SD, severe dysplasia; SCC, squamous cell carcinoma; AUC, area under the ROC curve.

To clarify the association between the NBI MOI and the progression of OSCC, NBI and quantitative analysis were performed on the lingual tissues of mice at distinct pathological stages of 4NQO-induced OSCC, including HC mucosa, MiD, MoD, SD, and squamous cell carcinoma (SCC). Representative NBI images revealed marked visual differences in the optical signals of the lingual mucosa between the control group and the model group at different lesion stages (Figure 3). Further quantitative analysis of the MOI demonstrated a continuous decreasing trend in the lingual mucosa of mice as the severity of malignancy progressed (Figure 4A). Specifically, the MOI of the lingual mucosa in the HC group was the highest, reaching 167.3 ± 10.05 a.u., followed by 126.4 ± 11.74 a.u. in the MiD group, 99.22 ± 4.801 a.u. in the MoD group, and 70.77 ± 4.019 a.u. in the SD group. When the lesions progressed to OSCC, this index dropped to the lowest level (48.67 ± 10.07 a.u.), indicating a significant negative correlation between the NBI optical intensity and lesion severity (*p* < 0.001).

To verify the statistical reliability of the above trend, the Shapiro–Wilk test was first performed to confirm that the data of all groups conformed to a normal distribution, followed by one-way ANOVA. The results showed a highly significant difference in MOIs among the five groups (HC group and model groups at different lesion stages) (*F* (4, 29) = 209.3, *p* < 0.0001). In addition, the Brown–Forsythe test confirmed the homogeneity of variances among groups (*p* = 0.817), which satisfied the application conditions of ANOVA. The R^2^ of the regression model reached 0.967, suggesting that changes in optical signal intensity could explain 96.7% of the variance in pathological lesion stages, indicating a strong correlation between this quantitative metric and OSCC pathological progression. However, given the relatively small sample size in this preclinical model, this high R^2^ should be interpreted cautiously as it may reflect robust within-model fitting rather than broad external generalizability. Independent validation using larger sample cohorts is required to further verify this correlation.

To identify the specific differences between groups, Dunnett’s multiple comparison test was further conducted with the lingual mucosa of normal mice as the reference control. The results showed that the differences in MOI between all lesion groups and the HC group were extremely significant (all *p* < 0.001), suggesting that the NBI MOI could effectively distinguish normal mucosa from lesion tissues at different stages. At the end of the experiment, histopathological examination was performed on all imaged regions, and the results were highly consistent with those of the NBI analysis. Regions with decreased MOI all exhibited varying degrees of epithelial dysplasia, invasive growth, and stromal reaction, while no evidence of malignant lesions was found via histological examination in regions where the optical intensity remained within the normal range, thereby confirming the accuracy of NBI MOI in reflecting pathological changes.

An ROC curve was further constructed to quantify the diagnostic efficiency (Figure 4B). The AUC of NBI MOI for distinguishing early-stage (MiD) from advanced-stage lesions (MoD + SD + SCC) was 0.989 (95% CI: 0.954–1.000), indicating excellent diagnostic performance. The optimal cutoff value, determined based on the maximum Youden index, was 103.5 a.u., yielding a sensitivity of 93.3% (for identifying advanced-stage lesions) and a specificity of 83.3% (for identifying early-stage lesions). Stratified 5-fold cross-validation was subsequently conducted to verify the stability of diagnostic performance, yielding a cv-AUC of 0.951 (95% CI: 0.892—0.998). When applying the same cut-off value of 103.5 a.u. from the primary ROC analysis, the cross-validated sensitivity and specificity were 96.7% and 80.0%, respectively. These results demonstrate that NBI MOI could effectively differentiate between early and advanced lesions in this model.

### 3.3. NBI Imaging Enables Accurate Identification of Syngeneic Tumor Lesion Boundaries with a Marked Reduction in Optical Intensity

In the syngeneic graft tumor model, eight OSCC syngeneic graft-bearing mice (syngeneic tumor group) and eight healthy mice (HC group) were enrolled. The morphological characteristics and NBI optical manifestations of the dorsal tongue mucosa between the two groups were compared using NBI.

**Figure 5:**
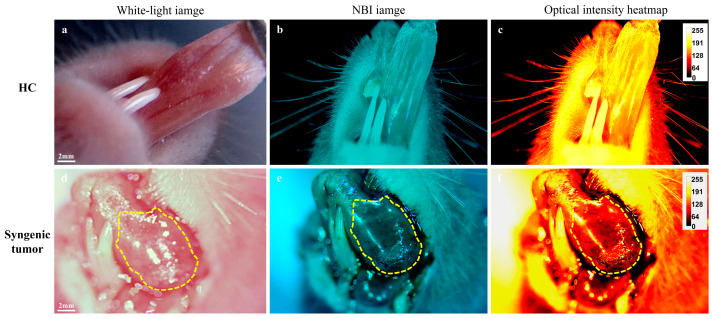
Representative macroscopic, NBI, and optical density heatmap images of dorsal tongue mucosa from HC and syngeneic tumor-bearing mice. (**a**–**c**) White-light, NBI, and corresponding optical density heatmap images of normal dorsal tongue mucosa in the HC group, showing a smooth mucosal surface with uniform optical signal distribution. (**d**–**f**) White-light, NBI, and corresponding optical density heatmap images of the syngeneic tumor group. Yellow dashed outlines demarcate the tumor lesion boundary. The tumor presents as a dark purple, round raised lesion, accompanied by markedly reduced optical density within the lesion area, whereas the surrounding normal mucosa maintains consistent optical intensity. Scale bar = 2 mm. NBI, narrow-band imaging; HC, healthy control.

Representative images (Figure 5) showed that the dorsal tongue mucosa of mice in the HC group exhibited a uniform color and smooth surface under white light, without abnormal protuberances or lesions. In the corresponding NBI images, the normal oral mucosa exhibited a globally homogeneous optical signal distribution with consistent optical intensity, which clearly delineated its anatomical boundaries. In contrast, the dorsal tongue mucosa of mice in the syngeneic tumor group showed localized dark-purple lesions under white light, appearing as round-like protuberances with distinct margins from adjacent normal tissue. In NBI images, these lesions presented as areas with markedly reduced optical intensity. Notably, regions with decreased optical density precisely overlapped with lesion areas identified under white light, whereas the perilesional normal mucosa retained uniform optical signals, generating clear visual contrast.

**Figure 6:**
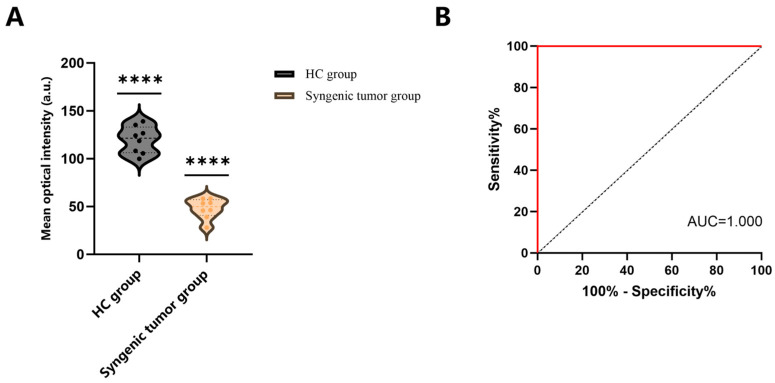
Quantitative comparison and diagnostic performance of MOI between HC and syngeneic OSCC tumor-bearing mice. (**A**) Violin-box plots showing significantly decreased MOI values in the syngeneic tumor group compared with the HC group. Data are presented as the mean ± SD, analyzed via an unpaired two-sample Student’s *t*-test (**** *p* < 0.0001). (**B**) ROC curve analysis of MOI for discriminating syngeneic tumor lesions from normal oral mucosa. The red line represents the ROC curve of MOI. AUC = 1.000 (95% CI: 1.000–1.000). At the optimal cut-off value, the sensitivity was 100.0% and the specificity was 87.5%. Stratified 5-fold cross-validation further validated diagnostic stability, with a cv-AUC = 0.984 (95% CI: 0.941–1.000). HC, healthy control; OSCC, oral squamous cell carcinoma; AUC, area under the ROC curve.

To quantify the differences in optical characteristics between the two groups, the MOI of the dorsal tongue mucosa was measured, and statistical analyses were performed using GraphPad Prism 10 software. Normality tests confirmed that all data met the requirements for parametric tests. Results of the unpaired two-sample Student’s *t*-test indicated that the MOI of the dorsal tongue mucosa in the HC group was 119.7 ± 14.20 a.u., which was significantly higher than the baseline level (theoretical mean = 0) (*t* = 23.83, *df* = 7, *p* < 0.0001). The MOI of the lesioned dorsal tongue mucosa in the syngeneic tumor group was 47.85 ± 10.44 a.u., also significantly higher than the baseline level (*t* = 12.96, *df* = 7, *p* < 0.0001). These findings demonstrated that both groups of dorsal tongue mucosa exhibited stably detectable specific optical signals. Violin plots were generated using GraphPad Prism 10 to visually present the data distribution and differences between the two groups (Figure 6A). The results showed that the MOI of the lesioned dorsal tongue mucosa in the syngeneic tumor group was significantly lower than that in the HC group (*p* < 0.001). These data confirmed a significant correlation between MOI and the pathological status of the dorsal tongue mucosa, suggesting that this indicator can effectively distinguish OSCC syngeneic graft tissues from the normal oral mucosa.

We constructed an ROC curve to evaluate the diagnostic ability of the MOI in distinguishing the syngeneic tumor-bearing oral mucosa from the normal mucosa (Figure 6B). The AUC reached 1.000 (95% CI: 1.000–1.000, *p* = 0.0008), indicating perfect diagnostic performance in the primary dataset. At the optimal cutoff value of 102.7 a.u., the sensitivity and specificity were 100.0% and 87.5%, respectively. Stratified 5-fold cross-validation was further performed to avoid overfitting, yielding a cv-AUC of 0.984 (95% CI: 0.941–1.000) and verifying the favorable diagnostic stability. Collectively, these findings suggest that the NBI-derived MOI may serve as a reliable quantitative marker for identifying OSCC lesions in this syngeneic tumor mouse model.

## 4. Discussion

Through multi-dimensional validation using clinical samples, 4NQO-induced OSCC models, and OSCC syngeneic tumor models, this study systematically clarifies the diagnostic and monitoring value of NBI MOI in the “normal mucosa—dysplasia—carcinogenesis” progression of OSCC.

The key finding of this study is that NBI-detected MOI could serve as a reliable, quantitative imaging biomarker for distinguishing the normal oral mucosa, OLK, and OSCC tissues. This conclusion was consistently validated across multiple models: in clinical samples, the MOI showed a significant progressive decrease from the HC group (129.6 ± 7.386 a.u.) and OLK group (93.55 ± 9.066 a.u.) to the OSCC group (55.69 ± 14.98 a.u.) (*p* < 0.001). In the 4NQO-induced model, the MOI of the dorsal tongue mucosa was highest in HC mice (167.3 ± 10.05 a.u.) and decreased to the lowest level (48.67 ± 10.07 a.u.) at the OSCC stage, with the indicator explaining 96.65% of the variation in lesion stages (R^2^ = 0.967). In the syngeneic tumor model, the MOI of the lesioned dorsal tongue mucosa (47.85 ± 10.44 a.u.) was significantly lower than that of the HC group (119.7 ± 14.20 a.u.) (*p* < 0.001), and the hypointense area precisely coincided with the lesion area observed under white light.

This consistent trend across different models supports the reliability of our analytical approach and provides preliminary preclinical evidence for the potential application of NBI as an auxiliary tool for early-stage OSCC screening or intraoperative guidance [18]. Clinically, the kappa value reflecting consistency between optical intensity measurements and pathological diagnosis exceeded 0.75, suggesting that NBI-derived quantitative metrics may help complement the limitations of conventional invasive pathological biopsy. Notably, the high apparent diagnostic performance, indicated by raw AUC values up to 0.989, should be interpreted with caution, as small-sample analyses are prone to optimism bias and overfitting. To mitigate such bias, 5-fold cross-validation was implemented in all ROC analyses to generate more conservative and generalizable cv-AUC values. Collectively, these findings demonstrate promising diagnostic potential for NBI-derived optical intensity, though further validation in larger independent cohorts and multicenter studies is required prior to clinical translation. Moreover, the high R^2^ = 0.967 obtained in the 4NQO-induced mouse model suggests a strong linear association between decreased optical signal intensity and pathological progression. Owing to the relatively limited sample size, however, this correlation should be interpreted prudently, as small datasets may inflate the risk of overfitting.

The consistent progressive decline in NBI-derived MOI from the normal oral mucosa, through epithelial dysplasia, to OSCC was validated across clinical samples, 4NQO-induced mouse models, and syngeneic tumor models in the present study. This quantitative association provides reproducible experimental evidence supporting the potential utility of this metric for lesion stratification and longitudinal monitoring. Although the biological mechanisms underlying this signal reduction remain unconfirmed in our current work, we propose plausible mechanistic hypotheses based on existing literature regarding key pathophysiological alterations during oral carcinogenesis. First, metabolic reprogramming in dysplastic and malignant cells may alter endogenous chromophore levels such as NAD(P)H and FAD, which modulate light-reflective properties [19]. Second, remodeling of the extracellular matrix, including collagen degradation and architectural disorganization, may attenuate tissue optical signals [20]. Furthermore, aberrant submucosal microvascular changes—including neovascularization, vascular tortuosity, and increased vascular permeability—may increase hemoglobin-mediated light absorption, thereby further reducing detectable NBI signal intensity [21]. Collectively, these multifactorial tissue alterations may account for the gradual decrease in the optical intensity observed in NBI imaging. Importantly, direct experimental validation, such as metabolic profiling, quantitative collagen assessment, or high-resolution vascular imaging, was not performed in this study; accordingly, these mechanistic interpretations remain hypothetical and require further empirical verification.

Unlike previous qualitative studies that rely on lesion morphological assessments, the present study quantifies MOI to minimize subjective bias from visual inspection. This quantitative parameter is closely associated with intrinsic pathophysiological alterations within oral mucosal lesions, which may help improve the reliability and reproducibility of the diagnostic evaluation [22]. Furthermore, NBI-derived optical density mapping demonstrated favorable performance for lesion identification: regions with reduced optical intensity based on mucosal lesions closely overlapped with dark-purple areas under NBI, enabling the clear demarcation of lesion boundaries and providing visual guidance for targeted pathological sampling [23,24]. This multimodal imaging approach integrates the strengths of NBI for visualizing the microvascular architecture and optical intensity measurements for reflecting the tissue pathological status, facilitating a comprehensive lesion evaluation. Compared with single-modal imaging, this combined strategy offers potential clinical utility and may provide insights for optimizing OSCC diagnostic workflows [7,25].

Notably, MOI can be readily integrated into routine clinical workflows for real-time implementation. During standard oral endoscopic examinations, NBI imaging is acquired under dim ambient light. The region of interest covering lesions and the adjacent normal mucosa can be rapidly delineated via built-in or post-processing software, with the MOI calculated automatically within seconds to deliver immediate quantitative readouts for real-time discrimination of the normal mucosa, dysplasia, and OSCC. This non-invasive procedure requires no extra reagents or contrast agents and does not prolong the examination duration, supporting its practical use as a quantitative biomarker for screening, risk stratification, intraoperative margin assessment, and postoperative surveillance in daily clinical practice.

With ongoing advances in artificial intelligence and medical image analysis, this visual-quantitative integrated framework could be further refined into an automated, intelligent, non-invasive diagnostic platform [26,27]. The construction of large-scale, multicenter, pathologically annotated NBI image datasets may support deep-learning-assisted workflows for automated lesion detection, region-of-interest segmentation, MOI quantification, and pathological risk grading, which may facilitate real-time, operator-independent non-invasive screening and risk stratification for OSCC and its precancerous lesions in future clinical settings [28,29].

Despite these promising findings, several limitations of the present study should be acknowledged. First, all human clinical samples were retrospectively collected from a single center, which may introduce selection bias and limit the generalizability of our results. Prospective multicenter studies with larger sample sizes are therefore needed to validate the external validity of our observations. Second, longitudinal cohort studies are required to clarify the dynamic changes in the MOI during the progression of human OSCC.

## 5. Conclusions

Through multi-dimensional validation using human clinical specimens and animal models, this study demonstrates that the quantitative assessment of MOI under NBI enables the identification of pathological alterations along the “normal oral mucosa-dysplasia-carcinogenesis” sequence of OSCC, with diagnostic outcomes well correlated with histopathological findings.

In conclusion, the NBI-derived MOI biomarker shows promising clinical potential for the early detection, longitudinal monitoring, and therapeutic assessment of OSCC. Characterized by non-invasiveness, good repeatability, and quantitative readouts, this parameter may overcome the limitations of conventional pathological evaluations. Following further multicenter validation, technical optimization, and clinical translational research, NBI-based MOI may serve as a feasible non-invasive adjunct marker for early OSCC screening, particularly in high-risk populations, including patients with OLK. Standardized detection workflows may be integrated into clinical diagnostic pathways to support dynamic monitoring and risk stratification, potentially improving patient prognosis and facilitating precision oncology practice for OSCC.

## Data Availability

The datasets used and analyzed in this study are available from the corresponding authors upon reasonable request.

## Supplementary Materials

The following supporting information can be downloaded at: https://www.mdpi.com/article/10.3390/biomedicines14061234/s1.

## Abbreviations

The following abbreviations are used in this manuscript:

4NQO	4-nitroquinoline 1-oxide
a.u.	arbitrary units
AUC	Area under the ROC curve
CI	Confidence interval
cv-AUC	cross-validated area under the curve
H&E	Hematoxylin and eosin
HC	Healthy control
MOI	Mean optical intensity
MiD	Mild dysplasia
MoD	Moderate dysplasia
NBI	Narrow-band imaging
OLK	Oral leukoplakia
OSCC	Oral squamous cell carcinoma
ROC	Receiver operating characteristic
ROI	Region of interest
SCC	Squamous cell carcinoma
SD	Severe dysplasia
